# Elder Abuse Multidisciplinary Teams: Describing and Classifying a Key Collaborative Resource for APS Workers

**DOI:** 10.1093/geroni/igaa057.2439

**Published:** 2020-12-16

**Authors:** Zachary Gassoumis, Gerson Galdamez, Julia Rowan, Kathleen Wilber

**Affiliations:** University of Southern California, Los Angeles, California, United States

## Abstract

Elder abuse multidisciplinary teams (MDTs) are a key resource when APS workers address their most complex cases. MDTs promote coordination and information sharing, and provide access to highly specialized input and problem-solving from legal, health, social service, and financial fields. This paper characterizes the range of elder abuse MDTs across the U.S. We identified 324 MDTs in the U.S., which most frequently addressed cases of financial exploitation (90.8%), physical abuse (83.6%) and neglect (81.6%). Based on a follow-up survey, latent class analysis was used to determine closeness of a subset (n=91) to the elder abuse forensic center model, which has received much evaluation and policy attention. Twenty-six showed strong similarity to forensic centers, with 24 others showing partial similarity. Coupled with observations from site visits to 4 teams, findings can guide the development and evaluation of elder abuse MDTs to foster better interdisciplinary collaboration for APS workers. Part of a symposium sponsored by Abuse, Neglect and Exploitation of Elderly People Interest Group.

